# Characterizing and Modeling Citation Dynamics

**DOI:** 10.1371/journal.pone.0024926

**Published:** 2011-09-22

**Authors:** Young-Ho Eom, Santo Fortunato

**Affiliations:** Complex Networks and Systems Lagrange Laboratory, Institute for Scientific Interchange, Torino, Italy; University of Maribor, Slovenia

## Abstract

Citation distributions are crucial for the analysis and modeling of the activity of scientists. We investigated bibliometric data of papers published in journals of the American Physical Society, searching for the type of function which best describes the observed citation distributions. We used the goodness of fit with Kolmogorov-Smirnov statistics for three classes of functions: log-normal, simple power law and shifted power law. The shifted power law turns out to be the most reliable hypothesis for all citation networks we derived, which correspond to different time spans. We find that citation dynamics is characterized by bursts, usually occurring within a few years since publication of a paper, and the burst size spans several orders of magnitude. We also investigated the microscopic mechanisms for the evolution of citation networks, by proposing a linear preferential attachment with time dependent initial attractiveness. The model successfully reproduces the empirical citation distributions and accounts for the presence of citation bursts as well.

## Introduction

Citation networks are compact representations of the relationships between research products, both in the sciences and the humanities [1], [2]. As such they are a valuable tool to uncover the dynamics of scientific productivity and have been studied for a long time, since the seminal paper by De Solla Price [3]. In the last years, in particular, due to the increasing availability of large bibliographic data and computational resources, it is possible to build large networks and analyze them to an unprecedented level of accuracy.

In a citation network, each vertex represents a paper and there is a directed edge from paper 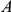 to paper 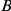 if 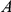 includes 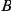 in its list of references. Citation networks are then directed, by construction, and acyclic, as papers can only point to older papers, so directed loops cannot be obtained. A large part of the literature on citation networks has focused on the characterization of the probability distribution of the number of citations received by a paper, and on the design of simple microscopic models able to reproduce the distribution. The number of citations of a paper is the number of incoming edges (indegree) 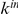 of the vertex representing the paper in the citation network. So the probability distribution of citations is just the indegree distribution 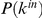. There is no doubt that citation distributions are broad, as there are papers with many citations together with many poorly cited (including many uncited) papers. However, as of today, the functional shape of citation distributions is still elusive. This is because the question is ill-defined. In fact, one may formulate it in a variety of different contexts, which generally yield different answers. For instance, one may wish to uncover the distribution from the global citation network including all papers published in all journals at all times. Otherwise, one may wish to specialize the query to specific disciplines or years. The role of the discipline considered is important and is liable to affect the final result. For instance, it is well known that papers in Biology are, on average, much more cited than papers in Mathematics. One may argue that this evidence may still be consistent with having similar functional distributions for the two disciplines, defined on ranges of different sizes. Also, the role of time is important. It is unlikely that citation distributions maintain the exact same shape regardless of the specific time window considered. The dynamics of scientific production has changed considerably in the last years. It is well known, for instance, that the number of published papers per year has been increasing exponentially until now [4]. This, together with the much quicker publication times of modern journals, has deeply affected the dynamics of citation accumulation of papers. Moreover, if the dataset at study includes papers published in different years, older papers tend to have more citations than recent ones just because they have been exposed for a longer time, not necessarily because they are better works: the age of a paper is an important factor.

So, the question of which function best describes the citation distributions is meaningless if one does not define precisely the set of publications examined. Redner [5] considered all papers published in Physical Review D up to 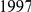, along with all articles indexed by Thomson Scientific in the period 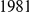
–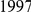, and found that the right tail of the distribution, corresponding to highly cited papers, follows a power law with exponent 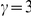, in accord with the conclusions of Price [3]. Laherrére and Sornette [6] studied the top 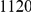 most cited physicists during the period 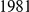
–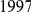, whose citation distribution is more compatible with a stretched exponential 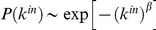, with 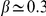. Tsallis and de Albuquerque [7] analyzed the same datasets used by Redner with an additional one including all papers published up to 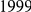 in Physical Review E, and found that the Tsallis distribution 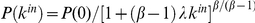, with 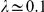 and 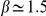, consistently fits the whole distribution of citations (not just the tail). More recently Redner performed an analysis over all papers published in the 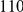 years long history of journals of the American Physical Society (APS) [8], concluding that the log-normal distribution
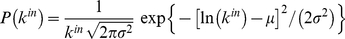
(1)is more adequate than a power law. In other studies distributions of citations have been fitted with various functional forms: power-law [9]–[14], log-normal [12], [15], [16], Tsallis distribution [17], [18], modified Bessel function [19], [20] or more complicated distributions [21].

In this paper we want to examine citation networks more in depth. We considered networks including all papers and their mutual citations within several time windows. We have performed a detailed analysis of the shape of the distributions, by computing the goodness of fits with Kolmogorov-Smirnov statistics of three model functions: simple power law, shifted power law and log-normal. Moreover, we have also examined dynamic aspects of the process of citation accumulation, revealing the existence of “bursts”, i.e. of rapid accretions of the number of citations received by papers. Citation bursts are not compatible with standard models of citation accumulation based on preferential attachment [22], in which the accumulation is smooth and papers may attract many cites long after publication. Therefore, we propose a model in which the citation attractiveness of a paper depends both on the number of cites already collected by the paper and on some intrinsic attractiveness that decays in time. The resulting picture delivers both the citation distribution and the presence of bursts.

## Results

### The distribution of cites

For our analysis we use the citation database of the American Physical Society (APS), described in Materials and Methods. We get the best fit for the empirical citation distributions from the goodness of fit test with Kolmogorov-Smirnov (KS) statistics [23]. The KS statistic 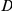 is the maximum distance between the cumulative distribution function (CDF) of the empirical data and the CDF of the fitted model:
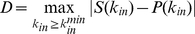
(2)Here 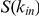 is the CDF of the empirical indegree 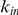 and 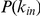 is the CDF of the model that fits best the empirical data in the region 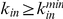. By searching the parameter space, the best hypothetical model is the one with the least value of 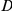 from the empirical data. To test the statistical significance of the hypothetical model, we cannot use the values of the KS statistics directly though, as the model has been derived from a best fit on the empirical data, rather than being an independent hypothesis. So, following Ref. [23] we generate synthetic datasets from the model corresponding to the best fit curve. For instance, if the best fit is the power law 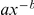, the datasets are generated from this distribution. Each synthetic dataset will give a value 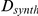 for the KS statistics between the dataset and the best fit curve. These 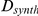-values are compared with 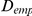, i.e. the 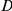-value between the original empirical data and the best fit curve, in order to define a 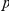-value. The 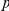-value is the fraction of 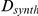-values larger than 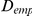. If 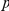 is large (close to 1), the model is a plausible fit to the empirical data; if it is close to 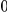, the hypothetical model is not a plausible fit. We applied this goodness of fit test to three hypothetical model distributions: log-normal, simple power law and shifted power law. The log-normal distribution for the indegree 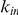 is given by
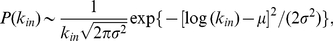
(3)the simple power law distribution by
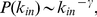
(4)and the shifted power law by
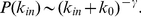
(5)We used 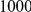 synthetic distributions to calculate the 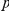-value for each empirical distribution.

**Figure 1:**
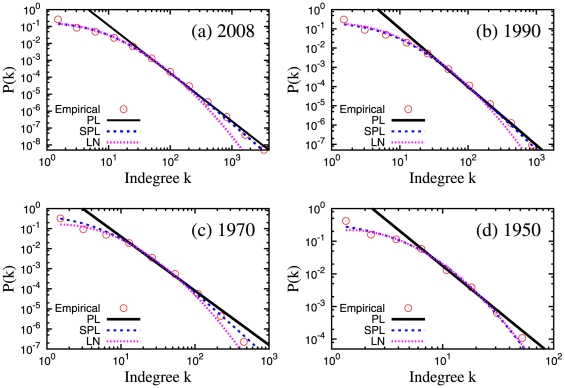
Empirical citation distributions and best fit model distributions obtained through the goodness of fit with Komolgorov-Smirnov statistics. PL: Power law. SPL: Shifted power law. LN: Log-normal.

Fig. 1 shows some fits for datasets corresponding to several time windows (see Materials and Methods). The detailed summary of the goodness of fit results is shown in Table 1. The simple power law gives high 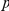-value only when one considers the right tail of the distribution (usually 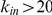). The log-normal distribution gives high 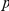-value for early years (before 1970) but after 1970 the p-value is smaller than 0.2. As shown in Figs. 1a and 1b, there is a clear discrepancy in the tail between the best fit log-normal distribution and the empirical distribution. The shifted power law distribution gives significant p-values (higher than 0.2) for all observation periods. The values of the exponent 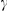 of the shifted power law are decreasing in time. The range of 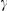 goes from 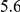 (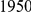) to 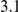 (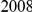).

**Table 1:** Summary of the results of the goodness of fit test with Kolmogorov-Smirnov statistic on the empirical citation distributions for three test functions: log-normal (LN), simple power law (PL) and shifted power law (SPL).

	1950	1955	1960	1965	1970	1975	1980	1985	1990	1995	2000	2005	2008
LN													
p-value	0.717	0.734	0.892	0.998	0.201	0.105	0.19	0.119	0.194	0.194	0.096	0.05	0.064
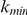	2	3	7	14	2	2	2	3	2	2	2	2	2
PL													
p-value	0.001	0.955	0.056	0.321	0.022	0.127	0.204	0.784	0.686	0.412	0.362	0.619	0.44
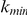	6	16	9	19	12	17	20	39	46	39	43	47	47
SPL													
p-value	0.832	0.777	0.49	1.00	0.943	0.958	0.49	0.728	0.909	1.00	0.797	0.989	0.99
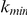	2	2	2	14	9	12	2	2	2	2	3	6	5

The fits are done for indegree larger than 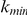, whose values are also reported in the table.

We conclude that the shifted power law is the best distribution to fit the data.

### The distribution of citation bursts

**Figure 2:**
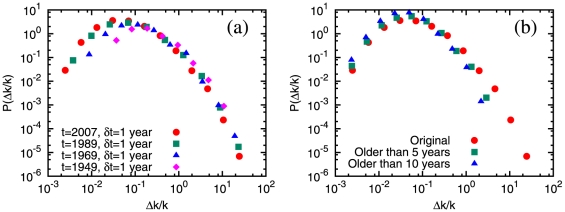
Distributions of citation burst size. (a) The four curves correspond to 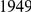, 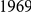, 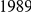 and 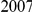, the observation window is 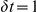 year. (b) Here the reference year is 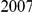, but the burst statistics is limited to the papers published until 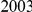 (squares) and 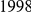 (triangles). For comparison, the full curve comprising all papers (circles, as in (a)) is also shown.

We now turn our attention to citation “bursts”. While there has been a sizeable activity in the analysis of bursty behavior in human dynamics [24]–[26], we are not aware of similar investigations for citation dynamics. We compute the relative rate 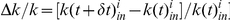, where 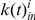 is the number of citations of paper 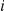 at time 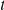. The distributions of 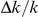 with 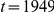, 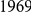, 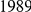, 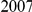 and 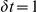 year are shown in Fig. 2a. They are visibly broad, spanning several orders of magnitude. Similar heavy tails of burst size distributions were observed in the dynamics of popularity in Wikipedia and the Web [27]. It is notable that the largest bursts take place in the first years after publication of a paper. This is manifest in Fig. 2b, where we show distributions derived from the same dataset as in Fig. 2a, but including only papers older than 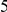 (squares) and 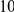 years (triangles): the tail disappears. In general, more than 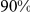 of large bursts (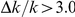) occur within the first 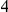 years since publication.

### Preferential attachment and age-dependent attractiveness

For many growing networks, cumulative advantage [28], [29], or preferential attachment [22], has proven to be a reliable mechanism to explain the fat-tailed distributions observed. In the context of citation dynamics, it is reasonable to assume that, if a paper is very cited, it will have an enhanced chance to receive citations in the future with respect to poorly cited papers. This can be formulated by stating that the probability that a paper gets cited is proportional to the number of citations it already received. That was the original idea of Price [30] and led to the development of the first dynamic mechanism for the generation of power law distributions in citation networks. In later refinements of the model, one has introduced an *attractiveness* for the vertices, indicating their own appeal to attract edges, regardless of degree. In particular, one has introduced the so-called *linear preferential attachment* [31], [32], in which the probability for a vertex to receive a new edge is proportional to the sum of the attractiveness of the vertex and its degree. In this Section we want to check whether this hypothesis holds for our datasets. This issue has been addressed in other works on citation analysis, like Refs. [13], [33].

**Figure 3:**
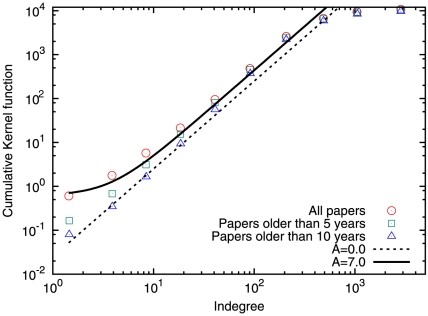
Cumulative kernel function of the citation network from 2007 to 2008. The continuous line is 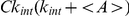 with 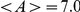, 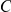 is a constant. The dashed line corresponds to the case without attractiveness (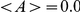).

We investigated the dependence of the kernel function 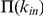 on indegree 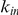 [34], [35]. The kernel is the rate with which a vertex 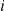 with indegree 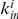 acquires new incoming edges. For linear preferential attachment the kernel is
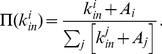
(6)In Eq. 6 the constant 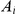 indicates the attractiveness of vertex 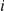. Computing the kernel directly for each indegree class (i.e. for all vertices with equal indegree 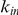) is not ideal, as the result may heavily fluctuate for large values of the indegree, due to poor statistics. So, following Refs. [34], [35], we consider the cumulative kernel 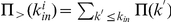, which, for the ansatz of Eq. 6, should have the following functional dependence on 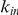


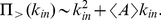
(7)In Eq. 7 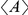 is the average attractiveness of the vertices. In order to estimate 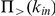, we need to compute the probability that vertices with equal indegree have gotten edges over a given time window, and sum the results over all indegree values from the smallest one to a given 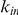. The time window has to be small enough in order to preserve the structure of the network but not too small in order to have enough citation statistics. In Fig. 3 we show the cumulative kernel function 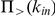 as a function of indegree for a time window from 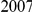 to 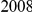. The profile of the curve (empty circles) is compatible with linear preferential attachment with an average attractiveness 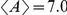 over a large range, although the final part of the tail is missed. Still, the slope of the tail, apart from the final plateau, is close to 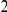, like in Eq. 7. Our result is consistent with that of Jeong et al. [34], who considered a citation network of papers published in Physical Review Letters in 1988, which are part of our dataset as well. We have repeated this analysis for several datasets, from 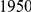 until 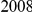, by keeping a time window of one year in each case. The resulting values of 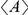 are reported in Table 2, along with the number of vertices and mean degree of the networks. The average value of the attractiveness across all datasets is 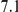. This value is much bigger than the average indegree in the early ages of the network like, for example, from 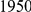 to 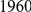. Hence, in the tradeoff between indegree and attractiveness of Eq. 6, the latter is quite important for old papers. In general, for low indegrees, attractiveness dominates over preferential attachment. As we see in Fig. 3, in fact, for low indegrees there is no power law dependence of the kernel on indegree.

**Table 2:** Statistics of the empirical citation networks: 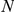 is the number of vertices in the network; 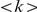 is the average indegree of the network; 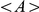 is the average attractiveness, determined from the tests of linear preferential attachment discussed in the text.

	1950	1955	1960	1965	1970	1975	1980	1985	1990	1995	2000	2005	2008
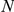	15880	23350	30996	42074	62382	85590	108794	138206	180708	238142	305570	386569	441595
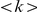	2.2	3.1	3.7	4.3	5.1	5.6	6.0	6.2	6.5	7.0	7.7	8.5	9.0
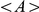	4.2	5.3	6.2	5.4	7.2	7.9	7.8	9.0	7.4	7.3	6.8	6.4	7.0

Finally we investigated the time dependence of the kernel. As shown in Fig. 3, when we limit the analysis to papers older than 5 years (squares) or 10 years (triangles), the kernel has a pure quadratic dependence on indegree in the initial part, without linear terms, so the attractiveness does not affect the citation dynamics. This means that the attractiveness has a significant influence on the evolution of the citation network only within the first few years after publication of the papers. The presence of vertex attractiveness had been considered by Jeong et al. as well [34].

### The model

We would like to design a microscopic model that reflects the observed properties of our citation networks. Preferential attachment does not account for the fact that the probability to receive citations may depend on time. In the Price model, for instance, papers keep collecting citations independently of their age, while it is empirically observed [33], [36], [37] that the probability for an article to get cited decreases as the age of the same article increases. In addition, we have seen that citation bursts typically occur in the early life of a paper. Some sophisticated growing network models include the aging of vertices as well [33], [37]–[40]. We propose a mechanism based on linear preferential attachment, where papers have individual values of the attractiveness, and the latter decays in time.

**Figure 4:**
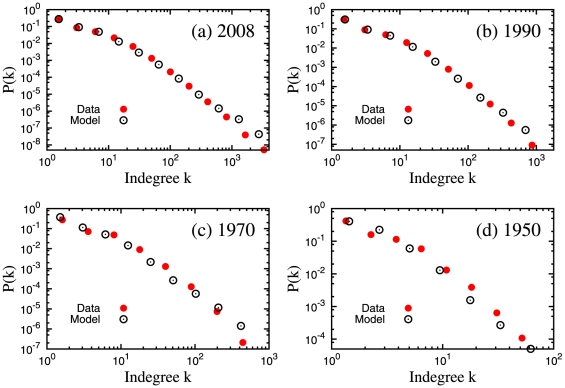
Comparison of the citation distributions from the empirical data and our model. For all cases, we used 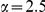 and 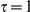 year. (a) For 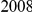, 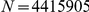, 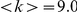. (b) For 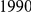, 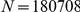, 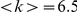. (c) For 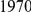, 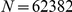, 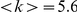. (d) For 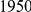, 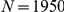, 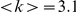. Here 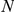 is the number of vertices/papers and 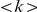 the average number of citations/indegree.

The model works as follows. At each time step 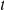, a new vertex joins the network (i.e., a new paper is published). The new vertex/paper has 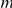 references to existing vertices/papers. The probability 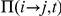 that the new vertex 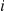 points to a target vertex 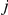 with indegree 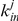 reads
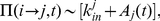
(8)where 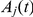 is the attractiveness of 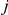 at time 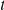. If 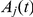 were constant and equal for all vertices we would recover the standard linear preferential attachment [31], [32]. We instead assume that it decays exponentially in time
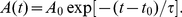
(9)In Eq. 9 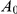 is the initial attractiveness of the vertex, and 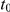 is the time in which the vertex first appears in the network; 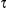 is the time scale of the decay, after which the attractiveness lowers considerably and loses importance for citation dynamics. Since citation bursts occur in the initial phase of a paper's life (Fig. 2b), when vertex attractiveness is most relevant, we expect that the values of the initial attractiveness are heterogeneously distributed, to account for the broad distribution of burst sizes (Fig. 2a). We assume the power law distribution
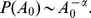
(10)We performed numerical simulations of the model with parameters obtained from the empirical data. We use 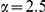, 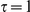 year and 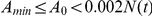 with 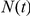 is the number of papers at time 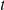. The upper bound represents the largest average indegree of our citation networks, expressed in terms of the number of vertices. The value of 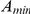 depends on the obtained value of the attractiveness from empirical data. We set 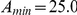 for most years, for 1950 we set 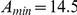, because 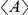 is smaller than 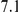. The result is however not very sensitive to the minimum and maximum value of 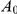. Fig. 4 shows the citation distributions of empirical data versus the model prediction. The model can reproduce the empirical distributions very well at different phases in the evolution of the APS citation network, from the remote 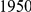 (panel d) until the very recent 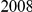 (panel a).

**Figure 5:**
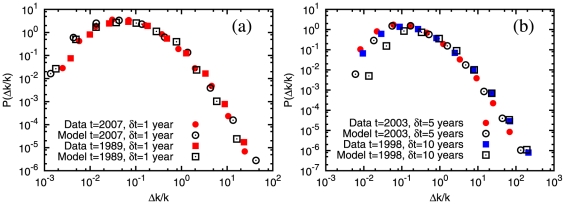
Comparison of the distributions of citation burst size from the empirical data and the model. The exponent 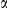 of the distribution of initial attractiveness is 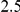, as in Fig. 4. (a) The reference years are 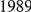 (squares) and 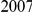 (circles), the observation window for the bursts is 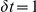 year in both cases. (b) Here the reference years are 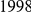 (squares) and 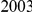 (circles) and the observation windows for the bursts are of 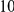 and 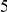 years, respectively.

The distributions of citation burst magnitude 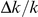 for the data and the model are shown in Fig. 5a. For a better comparison between data and model we “evolve” the network according to the model by starting from the structure of the empirical citation network at the beginning of the time window for the detection of the bursts. We stop the evolution after the observation time 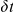 elapses. In Fig. 5a we consider 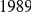 and 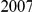, with a time window of 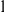 year for the burst detection. The model successfully reproduces the empirical distributions of burst size. In Fig. 5b we consider much longer observation periods for the bursts, of 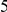 and 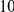 years. Still, the model gives an accurate description of the tail of the empirical curve in both cases.

## Discussion

We investigated citation dynamics for networks of papers published on journals of the American Physical Society. Kolmogorov-Smirnov statistics along with goodness of fit tests make us conclude that the best ansatz for the distribution of citations (from old times up to any given year) is a shifted power law. The latter beats both simple power laws, which are acceptable only on the right tails of the distributions, and log-normals, which are better than simple power laws on the left part of the curve, but are not accurate in the description of the right tails. We have also studied dynamic properties of citation flows, and found that the early life of papers is characterized by citation bursts, like already found for popularity dynamics in Wikipedia and the Web.

The existence of bursts is not compatible with traditional models based on preferential attachment, which are capable to account for the skewed citation distributions observed, but in which citation accumulation is smooth. Therefore we have introduced a variant of linear preferential attachment, with two new features: 1) the attractiveness decays exponentially in time, so it plays a role only in the early life of papers, after which it is dominated by the number of citations accumulated; 2) the attractiveness is not the same for all vertices but it follows a heterogeneous (power-law) distribution. We have found that this simple model is accurate in the description of the distributions of citations and burst sizes, across very different scientific ages. Moreover, the model is fairly robust with respect to the choice of the observation window for the bursts.

## Materials and Methods

Our citation database includes all papers published in journals of the American Physical Society (APS) from 1893 to 2008, except papers published in Reviews of Modern Physics. There are 3 992 736 citations among 414 977 papers at the end of 2008. The journals we considered are Physical Review (PR), Physical Review Letters (PRL), Physical Review A (PRA), Physical Review B (PRB), Physical Review C (PRC), Physical Review D (PRD), Physical Review E (PRE), Physical Review - Series I (PRI), Physical Review Special Topics - Accelerators and Beams (PRSTAB), and Physical Review Special Topics - Physics Education Research (PRSTPER). From these data, we constructed time-aggregated citation networks from 1950 to a year 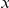, with 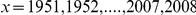.
